# Maternal hepatitis B and infant infection among pregnant women living with HIV in South Africa

**DOI:** 10.7448/IAS.17.1.18871

**Published:** 2014-05-22

**Authors:** Christopher J Hoffmann, Fildah Mashabela, Silvia Cohn, Jennifer D Hoffmann, Sanjay Lala, Neil A Martinson, Richard E Chaisson

**Affiliations:** 1 Center for TB Research, School of Medicine, Johns Hopkins University Baltimore, MD, USA; 2 Perinatal HIV Research Unit, University of the Witwatersrand, Johannesburg, South Africa; 3 Department of Pediatrics and Child Health, Chris Hani Baragwanath Academic Hospital, University of the Witwatersrand, Johannesburg, South Africa

**Keywords:** HIV, HBV, peripartum, transmission, vaccination, Africa, occult HBV

## Abstract

**Introduction:**

Globally, hepatitis B virus (HBV) infection is the leading cause of liver-related mortality. Newborn vaccination, maternal antiviral therapy and administering hepatitis B immune globulin shortly after birth can greatly reduce the risk of perinatal and infant infection. However, evidence-based policy regarding these interventions in Africa is hampered by gaps in knowledge of HBV epidemiology. We describe maternal chronic hepatitis B (CHB) prevalence and infant infection during the first year of life within a cohort of women living with HIV.

**Methods:**

We recruited and prospectively followed pregnant women living with HIV and their infants from prenatal clinics in an urban area of South Africa. Hepatitis B surface antigen, anti-hepatitis B surface antibodies and HBV DNA were assessed in all women. Hepatitis B testing was also performed at 6 and 52 weeks for all infants born to mothers with either positive surface antigen or detectable HBV DNA.

**Results:**

We enrolled 189 women with a median age of 29 years and median CD4 count of 348 cells/mm^3^. Fourteen had a positive surface antigen (7.4%), of which six were positive for “e” antigen. An additional three had detectable HBV DNA without positive surface antigen. One infant developed CHB and three others had evidence of transmission based on positive HBV DNA assays. HBV vaccinations were delivered at six weeks of life to all infants.

**Conclusions:**

Our findings highlight the risk of peripartum HBV transmission in this setting. Approaches to reducing this transmission should be considered.

## Introduction

Chronic hepatitis B (CHB) is the leading cause of liver disease and hepatocellular carcinoma worldwide [1,2]. CHB develops in 5 to 20% of adolescents and adults acutely infected with hepatitis B virus (HBV) and in the majority of infants who are perinatally infected. Horizontal transmission can be prevented by administration of a series of three hepatitis B vaccinations [3–5]. In addition, newborn babies who receive the first injection of the vaccine series within 24 hours of birth have a reduced risk of developing CHB from mother-to-child HBV transmission [6]. Because early administration of the vaccine reduces peripartum HBV infection, it is recommended by the World Health Organization and routinely provided in regions where peripartum transmission is common, such as in East Asia [7]. In other regions, such as in most parts of Africa, where CHB is also highly endemic, HBV transmission is thought to occur after the peripartum period [4,8–11]. Because of the apparent later acquisition, most African countries provide HBV infant vaccination with other routine immunizations at six weeks of age.

Potential reasons for a lower risk of vertical HBV transmission in Africa than in Asia is lower hepatitis B “e” antigen prevalence and lower HBV DNA levels among mothers with CHB [10,12,13]. However, there are few studies of vertical HBV transmission in Africa; several date from prior to the HIV epidemic and suggested low levels of vertical transmission [2]. HIV co-infection leads to a higher prevalence of “e” antigenemia and higher HBV DNA levels; possibly altering the dynamics of mother-to-child HBV transmission. In a cohort of pregnant women living with HIV from Soweto, South Africa, we describe the prevalence of CHB, characteristics of HBV infection in these women and mother-to-child HBV transmission events.

## Methods

We recruited pregnant women living with HIV, with and without tuberculosis disease, into a case-control prospective cohort study to assess maternal and infant outcomes. Recruitment occurred at prenatal care sections of 21 primary health clinics and 1 public tertiary care hospital in an urban area of South Africa. Inclusion criteria were maternal age ≥18 years, documented HIV-infection, estimated gestational age >13 weeks and residing in Soweto or surrounding areas. Follow-up of mother and infant continued until one year postpartum. All participants received prenatal, peripartum and postnatal care through the public-sector health system. Study-specific laboratory testing included HIV and hepatitis B specific assays, including hepatitis B surface antigen (HBsAg), anti-hepatitis B surface antibody (anti-HBs), and quantitative HBV DNA for all participants. Women and infants positive for HBsAg had testing for hepatitis B e antigen (HBeAg). Children born to women positive for HBsAg or with detectable HBV DNA had HBV specific testing at 6 and 52 weeks. Infants with a positive HBsAg test at six weeks had testing of stored whole blood from the third day of life for HBsAg and HBV DNA.

We used a single positive HBsAg result as a surrogate for CHB based on the assumption that the women participating in this study were probably infected earlier in life and acute HBV infection was not the cause of the positive assay. The formal definition of chronic HBV is two consecutive positive HBsAg tests six or more months apart. We defined occult hepatitis B as detectable HBV DNA among women without a positive HBsAg, consistent with other publications [14]. Perinatal transmission was defined by a third day of life sample positive for HBV DNA and negative for HBsAg. HBsAg and HBeAg testing was performed using the Abbott ARCHITECT system (Abbott Laboratories, Abbott Park, Illinois, USA) and quantitative HBV DNA was assayed using the COBAS AmpliPrep/COBAS TaqMan HBV Test with a lower limit of detection of 20 IU/mL (Roche Molecular Diagnostics, Basel, Switzerland). All laboratory testing occurred at an accredited commercial research laboratory. Written informed consent was obtained from all adult participants prior to study procedures. Approvals of the study and consent process were received from the Johns Hopkins University and the University of the Witwatersrand.

We compared proportions or medians in the cohort using non-parametric measures, due to a small number of women with CHB, using either the chi-square test or Wilcoxon rank sum test. We calculated exact 95% confidence intervals around our estimate of CHB prevalence using the binomial method. STATA version 13 (Stata Corporation, College Park, Texas, USA) was used for all analyses.

## Results

We recruited 189 pregnant women living with HIV with a median age of 29 years (interquartile range [IQR]: 26 to 31) and median enrolment CD4 count of 348 cells/mm^3^ (IQR: 232–471; Table 1). Prior to presenting in labour, the following prevention of mother-to-child-transmission of HIV approaches were used: 44 (23%) received zidovudine monotherapy, 20 (10%) received stavudine or zidovudine-based antiretroviral therapy (ART), 120 (63%) received tenofovir-based ART, and 5 (3%) were not recorded as having received any form of prevention-of-mother-to-child-transmission prior to presenting in labour. All women receiving zidovudine monotherapy or not receiving any form of prevention-of-mother-to-child-transmission received maternal single-dose nevirapine during labour. All infants received nevirapine prophylaxis postpartum. CHB was diagnosed in 14 women (7.4%; 95% CI: 4.3, 12). Of these women, 6/14 (43%) were positive for HBeAg and 9/14 (64%) had detectable HBV DNA. Three women with negative HBsAg assays also had detectable HBV DNA. Of the women negative for HBsAg, 47 (26%) had detectable anti-HBs antibodies resulting from either resolved HBV infection or immunization.

**Table 1:** Maternal characteristics overall and by HBsAg status

	Total cohort * n * (%) or median (IQR)	HBsAg-negative * n * (%) or median (IQR)	HBsAg-positive * n * (%) or median (IQR)
Pregnant women, number	189	175	14
Age, years	29 (26, 31)	29 (26, 31)	32 (30, 34)
CD4 count at enrolment, cell/mm^3^	348 (232, 471)	342 (232, 471)	364 (126, 459)
HIV RNA closest to delivery, log_10_ c/mL	2.1 (1.3, 3.4)	2.0 (1.3, 3.4)	2.7 (1.7, 3.4)
HIV RNA <400 c/mL			
No	108 (58)	97 (55)	11 (78)
Yes	81 (42)	78 (44)	3 (21)
Antiretroviral therapy			
Single dose NVP only	5 (3)	3 (2)	2 (14)
AZT monotherapy + s d NVP	44 (23)	41 (23)	3 (21)
D4T or AZT based ART^a^	20 (10)	20 (11)	0
TDF-based ART^a^	120 (63)	111 (63)	9 (64)
HBeAg			
Negative			8 (57)
Positive			6 (43)
Detectable HBV DNA			
No		165 (94)	5 (36)
Yes		3 (2)	9 (64)
Missing		7 (4)	0
HBV DNA, log_10_ IU/mL (among those with detectable HBV DNA)		1.7 (1.7, 2.2)	3.2 (2.3, 4.4)
Anti-HBs antibody			
Negative		128 (74)	
Positive		47 (26)	

a Also including lamivudine and efavirenz, nevirapine, or lopinavir/ritonavir. HBsAg, hepatitis B surface antigen; HBeAg, hepatitis B e antigen, NVP, nevirapine, AZT, zidovudine; D4T, stavudine; TDF, tenofovir disoproxil.

Postnatal data were available for 11 infants born to women with CHB and 3 born to women without CHB but with detectable HBV DNA. Three infants born to mothers with CHB were not tested because of the mothers moving away (two) or maternal death (one). Of these 14 infants tested for hepatitis B, 1 had CHB following peripartum infection. The infected infant had third day blood positive for HBV DNA at log_10_ 2.7 IU/ml but negative for HBsAg; at six weeks HBV DNA was log_10_ 6.8 IU/mL and HBsAg was positive. HBsAg remained positive and HBeAg was positive at 12 months at which time HBV DNA was not assayed. Three other infants appeared to have been infected based on detectable HBV DNA at either week 6 (one) or at 12 months (two) of life but we did not detect HBsAg in these infants (Table 2). The infant with peripartum CHB was born to a mother who received stavudine, lamivudine, and efavirenz starting five years prior to delivery and who had an HIV RNA below the limit of detection prior to delivery. However, she was HBeAg positive, and had a log_10_ HBV DNA of 8.3 IU/mL prior to delivery. Two of the mothers of infants with detectable HBV DNA were positive for both HBsAg and HBeAg; whereas the third had detectable HBV DNA, but was negative for both HBsAg and HBeAg. Two of these women received ART containing tenofovir, lamivudine, and efavirenz whereas one received zidovudine mono-therapy. All HBV exposed and unexposed infants received the initial dose of HBV vaccine when they were six weeks old; seven of the twelve mothers with CHB or occult HBV received tenofovir containing ART. None of the 14 infants were HIV-infected by 12 months post-partum.

**Table 2:** Maternal and infant test results for the 14 mothers-infant pairs with a positive maternal HBsAg test or detectable HBV DNA

Maternal						Infant	
	
Participant number	HBsAg/HBeAg	HBV DNA a , log10 IU/mL	HIV RNA a , c/mL	HBV active ART agents	Duration on ART, months b	HBsAg	HBV DNA, log10 IU/mL
1	+/ +	7.3	<20	3TC	60	Yes	6.8
2	+/ +	2.6	7435	TDF, 3TC	3	No	0
3	+/ +	3.8	605	TDF, 3TC	1	No	1.9
4	+/ +	4.9	365,673	TDF, 3TC	1	No	0
5	+/ +	0	<20	TDF, 3TC	3	No	0
6	+/ +	2.0	939	TDF, 3TC	0.5	No	3.5
7	+/ −	4.0	240	TDF, 3TC	3	No	0
8	+/ −	0	2660	TDF, 3TC	3	No	0
9	+/ −	2.1	535	None	2	No	0
10	+/ −	0	<20	TDF, 3TC	4	No	0
11	+/ −	0	87	TDF, 3TC	3	No	0
12	−/ −	2.2	<20	TDF, 3TC	22	No	0
13	−/ −	1.7	9250	None	0	No	0
14	−/ −	1.7	<20	TDF, 3TC	4	No	3.4

a Prepartum result closest to delivery;

b prepartum ART duration. HBV, hepatitis B virus; CHB, chronic hepatitis B; HBsAg, hepatitis B surface antigen; TDF, tenofovir; 3TC, lamivudine.

## Conclusions

In our study, we observed four vertically-infected infant HBV infections from fourteen CHB- infected pregnant women living with HIV – one infant with CHB and three with detectable HBV DNA without HBsAg. Unfortunately, because HBV vaccination at birth and HBV immune globulin are not routinely used in the public sector in South Africa, none of these infants received either intervention [6,15]. In addition, the mother of the infant who developed CHB did not receive tenofovir, an agent associated with HBV control [16]. She was receiving lamivudine, another agent with HBV activity, but her high HBV DNA level suggests that she had lamivudine resistant HBV, although we did not confirm this through molecular testing.

One infant developing CHB is insufficient to estimate incidence or draw broad conclusions on the risk of peripartum HBV infection. However 28% of infants born to mothers with CHB or occult HBV having some evidence of hepatitis B infection by the 12th month of life seems high and suggests that increased attention is warranted regarding peripartum and infant HBV transmission. An even higher incidence of perinatal transmission may have occurred had three-quarters of the women with CHB or occult HBV not received TDF-based ART [17].

Providing a first dose of HBV vaccine at birth (as is done for BCG and oral polio vaccine in South Africa and for hepatitis B in some Asian countries) may be an important step to reduce mother-to-child HBV transmission [7]. Future consideration of newborn vaccination needs to also be informed by cost. Although the protective effect of HBV vaccination among HIV exposed newborns is undefined, data on immunogenicity in HIV-exposed infants suggest an effective response [18,19].

Our finding of 7.4% of women with CHB is consistent with prior studies from the region [20,21]. Our proportion of 43% with “e” antigenemia is also consistent with other reports from HIV-coinfected populations in the region in which 38 to 53% of HBsAg positive participants were also positive for HBeAg [20–23]. Our finding of only 2% of women with occult hepatitis B (as defined by detectable HBV DNA with a negative HBsAg) is lower than reported from some studies that had sicker participants with lower CD4 counts [24,25]. The higher median CD4 count in our cohort and a higher fraction of participants receiving ART likely accounts for the low proportion of occult HBV as occult HBV appears to be partly a phenomenon of low CD4 count and lack of ART [26–28].

The strength of our study is that it is based on a prospective cohort recruited from routine prenatal care clinics with follow-up through the first year of an infant's life. However, there are several limitations. One is that we were missing data on infant HBV status for three infants born to women with CHB because the mothers moved locations or died. Another important limitation is the small number of mothers with CHB and the single HBV infected infant. Our results provide insight, but not sufficient data to draw broad conclusions, regarding HBV transmission epidemiology.

Additional data would be useful to describe the current transmission epidemiology in southern Africa – perinatal, infant and adult. To that end, additional mother-infant pair surveys would be valuable among mothers living with HIV and mothers not living with HIV. However, we believe that treatment of all women living with HIV with effective antiretrovirals during pregnancy, such as tenofovir, and instituting newborn HBV vaccination may be reasonable practice modifications while awaiting additional research findings.
